# Effect of Roller Pump Pulse in the Arterial Needle Area during Hemodialysis

**DOI:** 10.3390/diagnostics11112010

**Published:** 2021-10-29

**Authors:** Milos Kasparek, Ludmila Novakova, Jan Malik

**Affiliations:** 1 Department of Fluid Dynamics and Thermodynamics, Faculty of Mechanical Engineering CTU in Prague, Technická 4, 160 00 Prague, Czech Republic; M.Kasparek@fs.cvut.cz; 2 Department of Machines and Power Engineering, Faculty of Mechanical Engineering UJEP, Pasteurova 1, 400 96 Ústí nad Labem, Czech Republic; ludmila.novakova@ujep.cz; 3 3rd Department Internal Medicine and General University Hospital, First Medical Faculty, Charles University, U Nemocnice 1, 128 08 Prague, Czech Republic

**Keywords:** hemodialysis, wall shear stress, peristaltic pump, particle image velocimetry method

## Abstract

Vascular access is a lifeline for hemodialysis patients. Its lifetime is affected by many hemodynamic factors such as pressure, flow regime and wall shear stress. During hemodialysis, changes in hemodynamic parameters occur due to the flow from needles inserted into the vascular system. Primarily, there is a change in shear stress that affects the vascular wall. Pathological effects of high or low WSS are known. The effect of jet from a venous needle on hemodynamics parameters was studied, but the influence of the arterial needle on hemodynamics parameters is not sufficiently studied. To understand its possible effects, we performed in vivo and in vitro studies. Methods. In vivo experiment: The existence of flow reversal around the suction needle was visualized in a group of 12 randomly selected patients using ultrasound velocity profiling (Doppler ultrasonography) during hemodialysis. In vitro experiment: The flow field was measured using the stereo particle image velocimetry method (stereo PIV). Two regimes were studied. In the first regime, the fluid in the extracorporeal circuit was pumped by a peristaltic pump. In the second regime, the continuous pump was used in the extracorporeal circuit. The conditions were set to resemble those in vascular access during a hemodialysis session. Flow volume was set to 600 mL/min for vascular access and 200 mL/min for the extracorporeal circuit. Results. The main finding of this study was that the wall in the region of the arterial needle was stressed by backflow through the arterial needle. Since this was a variable, low-shear stress loading, it was one of the risk factors for the development of stenosis. Cyclic flow reversal was apparent in all of the included hemodialysis patients. The stereo PIV in vitro experiment revealed the oscillating character of wall shear stress (WSS) inside the model. High shear stress was documented upstream of the injection point of the arterial needle. An area of very low WSS was detected right behind the injection point during a pulse of the peristaltic pump. The minimal and maximal values of the WSS during a pulse of the peristaltic pump in the observed area were −0.7 Pa and 6 Pa, respectively. The distribution of wall shear stress with the continual pump used in the extracorporeal circuit was similar to the distribution during a pulse of the peristaltic one. However, the WSS values were continual; the WSS did not oscillate. WSS ranged between 4.8 Pa and 1.0 Pa.

## 1. Introduction

Hemodialysis is the most frequent method of renal replacement therapy needed for patients suffering from end-stage renal disease. An arteriovenous fistula is the preferred vascular access [1,2]. However, its lifespan is limited by the development of stenoses and thrombosis. Thrombosis is the most feared complication because it may lead to access abandonment. Although thrombosis usually follows the development of access stenosis, thrombosis occurs without any stenosis in some patients and its reason is not always revealed. Hemodynamic changes play a role in the development of both access stenosis and thrombosis. Flow jet in or around the dialysis needles affects the flow in the vascular access area and hence hemodynamic parameters [3]. A flow of any real fluid creates shear stress. Shear stress is the tangential stress due to the friction between moving particles of a fluid (fluid shear stress, FSS) or between moving particles of a fluid and the wall (wall shear stress, WSS) [4].

**Figure 1:**
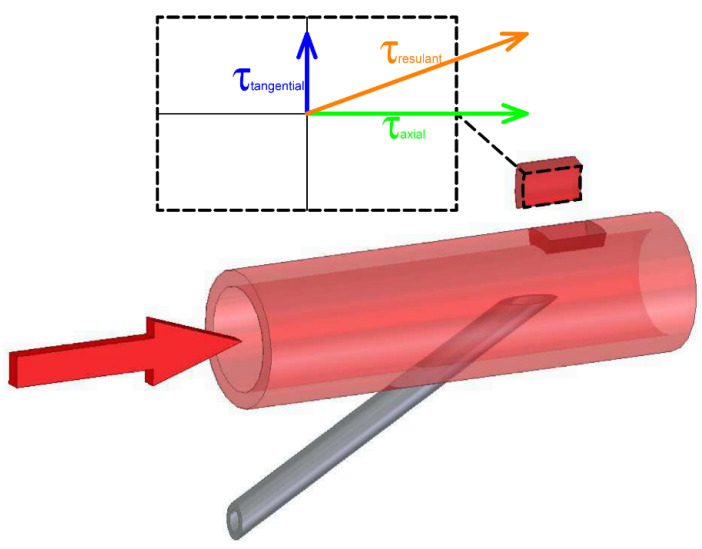
Schematic of the distribution of WSS components on the vessel wall. WSS has two components. These are tangential and axial components. The tangential component acts tangentially on the circumference of the vascular wall. The axial component of the WSS always acts in the direction of blood flow. The axial component of the WSS can therefore be used as an indicator of the direction of flow. If the value of the axial component becomes negative, then there is a backflow of blood.

The wall shear stress in multidimensional fluid flow can be divided into two components. The first component of the WSS acts in the direction of the axis of the tube in which the fluid is flowing ($\tau_{axial}$). The second component of the WSS acts in the tangential direction to the pipe wall and is referred to as tangential wall shear stress ($\tau_{tangential}$). The endothelial layer is primarily affected by two components of wall shear stress, namely the component in the direction of the tube axis ($\tau_{axial}$) and the component in the tangential direction to the tube wall ($\tau_{tangential}$). These two components influence the direction of endothelial cell stress. The direction of the endothelial cells is determined by the resultant of these two components of the WSS. Figure 1 shows a small portion of the study area with the components of tensile stress and their resultant ($\tau_{resulant}$). The resulting line shows the actual direction in which the endothelial cells will be directed. It can be seen from the figure that the direction of the resultant line is determined by the ratio of the sizes of the individual WSS components. The greater the difference in the sizes of the individual WSS components, the larger or smaller the ratio becomes and the more dominant component of the WSS results in a tilt of the resultant line. In the study area of the hemodialysis circuit, blood flows mainly in the direction of the vessel axis and therefore the dominant component of the WSS is in the direction of the axis ($\tau_{axial}$). This component always points in the direction of flow. Negative values are therefore indicative of reverse flow. The tangential component of the WSS only causes a tilt of the resulting WSS and does not provide information on the direction of flow. For this reason, the WSS component in the axial direction $\tau_{z}$ is used in this work to evaluate the influence of the vessel wall.

The component of wall tension in the z-axis direction can be expressed mathematically:

(1) $$\tau_{axial}=\mu \cdot \frac{\sigma w}{\sigma r}\;,$$

where $\tau_{axial}$ is the wall shear stress in the z-axis direction, *μ* is the dynamic viscosity and $\frac{\partial w}{\partial r}$ is the shear stress rate where *w* = velocity and *r* = radius.

Physiologically, wall shear stress is kept within a narrow band of values in the arteries and is responsible for arterial diameter control. There are two extreme conditions that connect shear stress and the incidence of vascular complications—values that are too high and values that are too low.

High values of wall shear stress can lead to endothelial denudation and, simultaneously, can lead to platelet and von Willebrand factor activation [5]. The high shear velocities are also connected with high shear stress. High shear stress causes the migration of erythrocytes (red blood cells) to the middle of the blood flow, which leads to a corresponding increase in the number of platelets in the proximity of the arterial wall [5]. Chronically low values of wall shear stress and its oscillating direction contribute to the incidence of vascular diseases, such as atherosclerosis and intimal hyperplasia [6,7,8,9]. Former studies linked these mechanisms only with low values of shear stress [10,11]. In a healthy person, common values of wall shear stress on the wall of venous walls are 0.1 to 0.6 Pa [12]. Values of WSS which oscillate under 0.1 Pa are dangerous for the venous wall.

The vascular flow in the proximity of the hemodialysis needles is influenced by the increased blood flow (jet) (venous needle) and by the suction of blood (arterial needle). The existence of the jet from the venous needle was analyzed in a study [13] where the jet was observed in a patient with an AVF during hemodialysis. An in vitro experimental study showed that the vessel wall was affected for up to 8 cm downstream of the tip of the needle [14]. A subsequent numerical study demonstrated that resulting WSS was elevated above normal physiological values in some areas [15]. Numerical studies [16,17] dealing with this issue have shown that the choroidal wall is affected by the suction of the arterial needle in its vicinity. However, these studies were performed with non-standard extracorporeal circuitry.

Most hemodialysis machines use blood peristaltic pumps. The principle of the peristaltic pump consists of the compression of a plastic blood tube against the pump wall by a roller placed on a rotating shaft. As the roller revolves, it pushes blood into the dialysis capsule. Peristaltic pumps normally have two or three rollers compressing the tubing. Two negative effects can be inferred from the principle of the peristaltic pump operation. The first is associated with the periodic compression of the tubing by the rollers, which causes oscillations in the blood flow. The second effect occurs at the beginning of the tube compression, when some of the blood is expelled from the compressed hose back into the vascular system via the arterial needle. We hypothesized that this temporary reversal of flow is transmitted via the “arterial” needle back to the vascular access and that it could cause significant changes in shear stress.

The aim of our study is to confirm the hypothesis of reverse flow through the arterial needle using experimental methods and to evaluate the effect on the vascular wall in the region of the arterial needle. Furthermore, the aim of the study is to compare the influence of the vascular wall when using a peristaltic pump in the excorporeal circuit with the influence of the vascular wall when using a continuous pump in the expoporal circuit in the given region of the arterial needle.

## 2. Materials and Methods

A two-stage study was conducted: 1. in vivo blood flow visualization in the area of the suction needle during hemodialysis using Doppler ultrasonography; 2. in vitro flow visualization and measurement of associated shear stress changes. After we confirmed our hypothesis as valid, we replaced the peristaltic pump with a continuous pump and studied its impact on the flow conditions.

### 2.1. In Vivo Study

We visualized the flow using ultrasound velocity profiling in 12 randomly selected patients with various types of native arteriovenous fistula during a hemodialysis session. The transportable Vivid Q (General Electric, Schenectady, NY, USA) ultrasound device was used for this purpose. It was equipped with a linear array high frequency probe with color Doppler mapping. Fistula blood flow was estimated and measured in the ipsilateral brachial artery by ultrasonography (with the use of cross-sectional area and time-averaged mean velocity). The patients were dialyzed using different devices and appropriate sets (Fresenius, Gambro and BBraun: 3 patients each). These measurements were part of a larger project that was confirmed by the ethical committee of the General University Hospital in Prague (on 23 May 2019 under the number 786/19).

### 2.2. In Vitro Study

**Figure 2:**
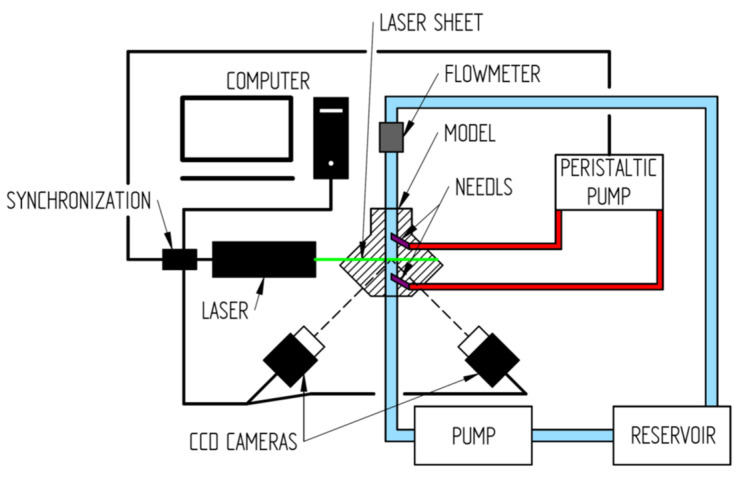
Schematic of experiment. The experimental setup has two circuits. The first circuit (blue) presents part of the vascular system with vascular access for hemodialysis. The second circuit (red) presents an extracorporeal circuit of hemodialysis. Two different pumps were used in this circuit; the first one was the peristaltic pump and the second one was the continual pump. In this experiment, the stereo PIV method, with two CCD cameras, was used for the measurement of flow in the area of the hemodialysis access.

Setup of experiment: We developed a model of arteriovenous access with two inserted needles. The boundary conditions were fitted to human data. The model consisted of two closed circuits—one represented the vascular access, the other the extracorporeal circuit of hemodialysis. They were connected by two “needles” corresponding to hemodialysis. Stable flow in the vascular access circuit was ensured by the continuous pump and was set to 600 mL/min. The flow in the extracorporeal circuit was set to 200 mL/min. The scheme of the experimental setup is shown in Figure 2. The model of the vascular access was a straight segment with the needles inserted at an angle of 30°, located at a distance of 60 mm. The diameter of the “outflow vein” was 6 mm and the needles were 1.6 mm thick. The extracorporeal circuit had two variants. The first one used a peristaltic pump for the motion of fluid, and the second used a continuous pump.

In vitro, we used the stereo particle imaging velocimetry (PIV) method for measuring the velocity fields [18,19]. The stereo PIV is an optical method, which allows all three velocity components in the measured plane to be obtained [20]. However, we can only obtain precise data by minimizing the optical deformations [21]. Therefore, it is necessary to use index-matching fluid that closely approximates the refractive index in the material of the vascular access model [22,23,24]. The working fluid was a solution of distilled water, sodium iodide and glycerine. This solution had similar dynamic viscosity to real blood (0.00431 Pa.s) and the refractive index was almost identical with the material of the vascular access model [25,26]. The viscosity of human blood is in the range of 0.0044 ± 0.0005 Pa.s. The viscosity of the working fluid was in the range of the viscosity of human blood. The velocity fields were measured in a set of cross sections. Cross sections were chosen to map the flow in the area around arterial needle. WSS was calculated from the flow characteristics that were recorded by PIV.

## 3. Results

### 3.1. In Vivo Experiment

We included 12 patients on chronic hemodialysis that were aged 71.8 ± 10.1 years: 6 males and 6 females. The etiology of kidney disease included type 2 diabetes mellitus of autosomal-dominant polycystic kidney diseases. They all had a native radiocephalic arteriovenous fistula as vascular access. Ipsilateral brachial artery blood flow was 1120 ± 362 mL/min and extracorporeal flow volume was set to 250–300 mL/min for the purpose of this study. Outflow vein diameter in the area of the punctures was 11 ± 3 mm. All needles were inserted in the direction of fistula blood flow (antegrade).

**Figure 3:**
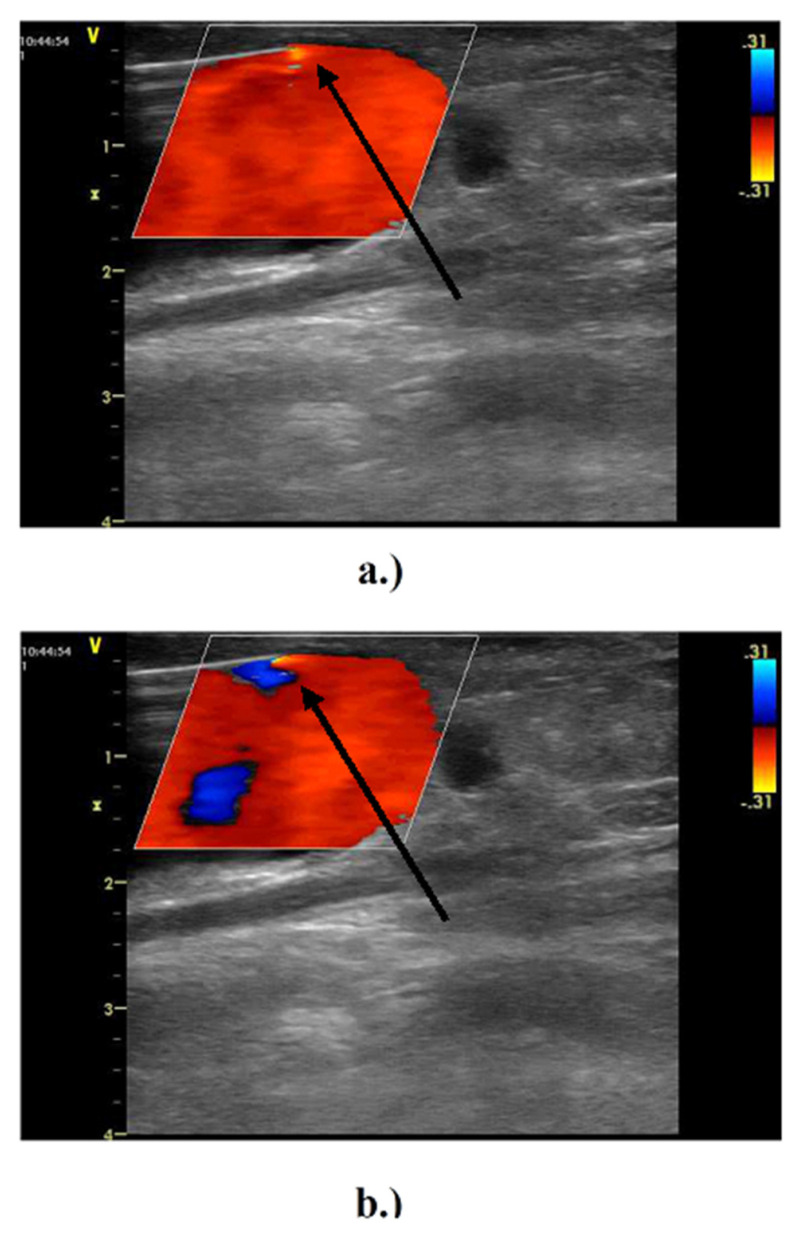
In vivo measurement of arterial needle blood flow during hemodialysis using Doppler ultrasound. Ipsilateral brachial artery blood flow was 1120 ± 362 mL/min and extracorporeal flow volume was set to 250–300 mL/min. Outflow vein diameter in the area of the punctures was 11 ± 3 mm. The prevailing direction of the flow (suction) at the needle tip (arrow) is highlighted using a lighter red color. (**a**,**b**) Flow reversal inside the vascular access at the suction needle due to the peristaltic pump action (small blue area, arrow).

Short-term cyclic flow reversal was evident at the entry into the suctional needle by ultrasonography (Figure 3) in all examined patients.

**Figure 4:**
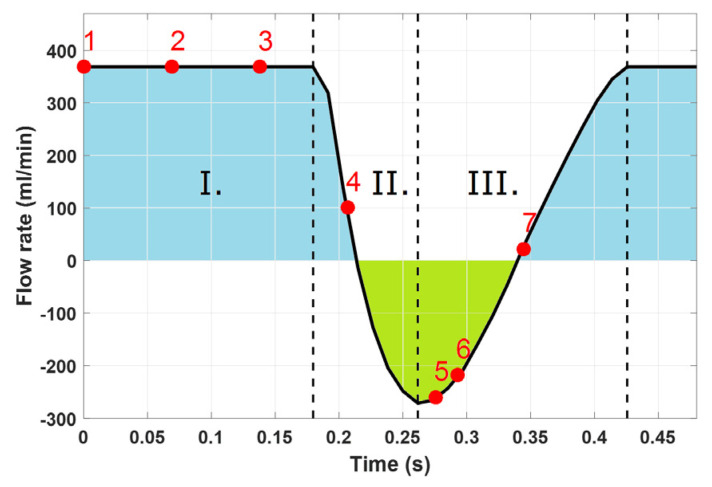
The pulse curve of the flow rate of the peristaltic pump in the extracorporeal circuit. The graph shows the volumetric flow rate pulse in the inlet of the peristaltic pump (arterial needle). The blue surface indicates the prograde velocity of the sucked blood, while the green surface represents the velocity of the flow reversal due to the peristaltic pump. The curve can be divided into three sections. Section 1 corresponds to constant suction. In Section 2, the deceleration of the intake flow is starting to take place, and in Section 3 the intake flow starts to accelerate again. Red points represent the points of pulse at which the flow of blood was measured using the PIV method in selected measured areas. At point 2, the interaction between the intake flow and vein wall is the strongest.

In Figure 4, the curve of the volumetric flow rate pulse on the inlet of the peristaltic pump is shown. This curve clearly shows the prograde and retrograde (reversal) velocities.

### 3.2. In Vitro

**Figure 5:**
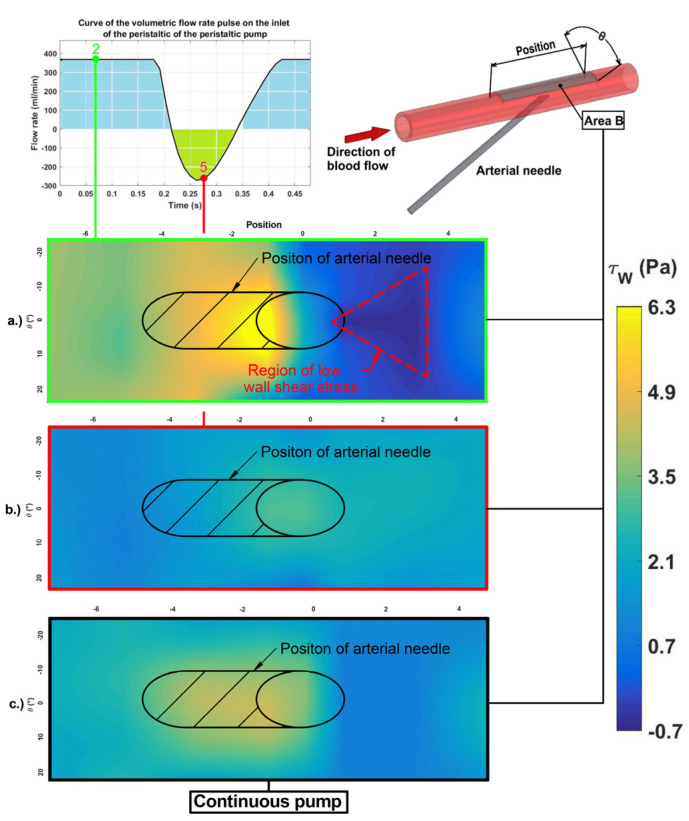
Wall shear stress distribution along the monitored area. The top right part of the figure shows part of the vascular access with a monitored area of WSS (grey, Area B). The position of the monitored area covers the area around the arterial needle. Graphs (**a**–**c**) show WSS distribution along the vessel wall in Area B. At point 2 of the pulse in Graph (**a**) the area with higher values of WSS can be found upstream of the needle. Low WSS can be observed up to 3 mm downstream from the center of the needle. The WSS reaches negative values in this area. At point 5 of the pulse in Graph (**b**) the insignificant maximum of the WSS values shift above the center of the needle inlet. Graph (**c**) represents the distribution of the WSS for the continual pump in the extracorporeal circuit. The WSS distribution is similar to that of pulse point 2. The WSS does not achieve negative values.

The use of the peristaltic pump led to an apparent short-term flow reversal in the area of the arterial needle. An area of low oscillating WSS was detectable close to the arterial needle. In Figure 5 the distribution of WSS in the observed area is shown. This figure depicts the area of oscillating low wall shear stress behind the arterial needle, which can be dangerous for the vessel wall.

**Figure 6:**
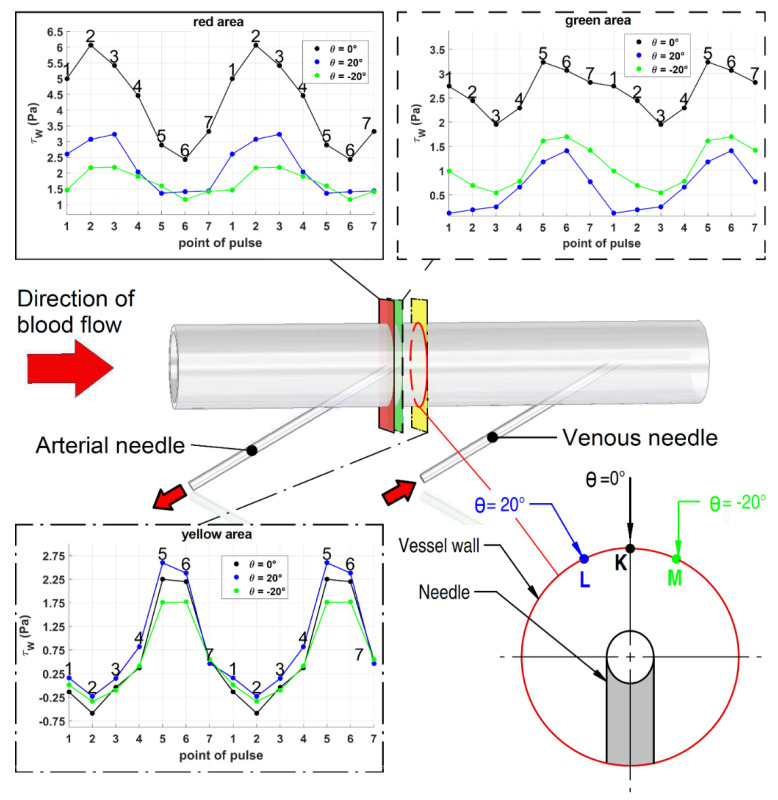
Wall shear stress for defined points K, L and M for all pulse points of the peristaltic pump in the selected measured areas. The bottom right part of the figure shows the monitored points locations on the inner surface of the vessel in a cross-sectional view. Three points (“K”, “L” and “M”) were chosen for three selected measured areas (red, green and yellow). At these points, WSS was monitored during pulse of the peristaltic pump. Red area (**upper left** graph): a clear oscillation character of WSS can be seen for all monitored points. Directly above the needle (black line) the WSS values for all pulse points are higher than for the other two observed angles. This is caused by the drawn blood flow of the needle affecting the flow in the artery. A maximum value of 6 Pa was reached at pulse point 2 for the angle θ = 0°. Green area (**upper right** graph): the values of WSS for the angle θ = 0° are significantly higher than for the other two angles. For all measured areas, a phase shift of the maximum of the wall tangential stress compared to the red area can be observed. This phase shift is caused by a drop in volumetric flow rate due to a significant amount of blood being drawn from the vascular access. At pulse points 5 and 6, no blood is being drawn and a portion of the drawn blood volume is returned to the artery through the arterial needle. The maximum WSS of 3.25 Pa was observed for the angle θ = 0°. Yellow area (**bottom** graph): the curve of WSS is similar for all measured points. A clear drop to negative values of WSS is observed at pulse point 2 for all curves.

In Figure 6, wall tangential stress for the defined points for all pulse points of the peristaltic pump in the selected measured areas are shown. WSS had a maximal value of 2.75 Pa and a minimal value of −0.7 Pa in the inspected area (behind the arterial needle). In the area before arterial needle, where the maximal influence of flow of the sucked blood was, the maximal WSS value was 6.1 Pa and the minimal WSS value was 2.5 Pa—these values are ten times higher than the physiological ones.

The replacement of the extracorporeal circuit peristaltic pump by a continuous pump led to a significant change in the flow behavior. Oscillating behavior of WSS was not observed in the measurement area and minimal WSS did not reach negative values. Minimal WSS behind the arterial needle (the dangerous area) was 1 Pa. In the area before the arterial needle the minimal WSS was 4.8 Pa.

## 4. Discussion

Our study revealed several significant findings of the behavior of the flow in the vascular access during hemodialysis using peristaltic pumps in the extracorporeal circuit. The in vivo experiment confirmed that the peristaltic pump influenced the flow of blood in the area of the arterial needle according to our assumptions. The in vitro experiment confirmed that the use of the peristaltic pump in the extracorporeal circuit leads to profound flow changes with oscillating low WSS inside the vascular access. The consequence includes reaching pathological values of WSS. 

Observed values of WSS around the arterial needle are known to cause endothelial activation and to increase endothelial permeability. In a healthy person, common values of WSS for venous walls are 0.1 to 0.6 Pa [12]. The replacement of the peristaltic pump by a continuous pump led to non-oscillation flow. However, the flow wake occurred in both types of pumps and was caused by the needle itself. Flow wake is the space behind the arterial needle where the velocity of the blood considerably decreases.

The flow characteristics around the arterial needle were studied by Fulker et al. in a numerical study [16], which focused on both dialysis needles; the tested direction of the arterial needle was both retrograde and antegrade. With the antegrade needle direction, there was a similar area of flow disruption as in our study. However, this study differs from ours not only by the methods (numerical vs. in vitro experiment) but also by another two factors. First, the shape of the peristaltic pump pulse was different to that which was used in the numerical study. It was based on a measurement carried out on a single patient during a hemodialysis, using Doppler ultrasonography [27]. Second, the setup of the extracorporeal circuit was not standard: the air detector was installed between the dialyzer and the venous needle as well as between the dialyzer and the arterial needle (non-standard). The air detector between the dialyzer and the arterial needle had a major dampening effect on the shape of the pulse of the peristaltic pump. Moreover, the temporary flow reversal was not revealed by Fulker´s study. The air detector partially absorbs reverse flow due to the principle of the working peristaltic pump. This leads to a decrease in the negative influence on the venous wall near the arterial needle.

Opposed to the peristaltic pump, the use of the continuous pump in the model was not associated with reaching pathological flow disturbances. The primary advantage of the peristaltic pump was that the blood was not in physical contact with the mechanical parts of the peristaltic pump. The next advantage of the peristaltic pump was the negative pressure, which the pump could generate on the intake side (real suction), and overpressure on the side of delivery. The main advantage of the continuous pump was that there were no oscillations during blood suction and thus no negative influence on the vascular wall. According to a study [28], the principle of the peristaltic pump can lead to damage to red blood cells. When using a continuous pump, the damage to red blood cells will be significantly reduced. Sterilizing of the continuous pump is possible, but it is necessary to exchange parts of continuous pump that are in contact with the blood. This would increase the price of its use in medical applications. A more reasonable solution is using a peristaltic pump, and adding a special hydraulic component to the extracorporeal circuit near the arterial needle, which would dampen the reverse flow caused by the peristaltic pump. The air detector can be used as a special hydraulic component, but the reversal flow damping is not ideal. It is possible to use a reverse throttle valve for more effective damping of reverse flow. This valve prevents reverse flow into the vascular access through the arterial needle. The disadvantage of this valve are shockwaves, which will be created due to the quickly closed valve. The shockwaves can have a negative influence on the blood. The next option is a Tesla valve, which reverses flow return to the correct direction of blood flow.

## 5. Conclusions

Our study documented that the peristaltic pump used in the extracorporeal circuit leads to potentially dangerous flow changes in the area of the arterial needle; this is caused by the pulse character of the peristaltic pump. This negative effect could be prevented either by using a continuous pump or inserting a special component in the set close to the arterial needle.

## Data Availability

Data supporting the reported results can be obtained upon request from the authors of the article: Milos Kasparek (M.Kasparek@fs.cvut.cz) and Jan Malik (jan.malik@lf1.cuni.cz).
